# Supplementary material for: Trends of Perinatal Stress, Anxiety, and Depression and Their Prediction on Postpartum Depression
Source: Int J Environ Res Public Health. 2021 Sep 3;18(17):9307. doi: 10.3390/ijerph18179307 (PMC8431252; doi:10.3390/ijerph18179307)
Supplement: Supplementary file 1 [file ijerph-18-09307-s001.zip › ijerph-1337945-supplementary.pdf]

Perceived Stress Scale

The questions in this scale ask you about your feelings and thoughts during the last month**. In each case, you will be asked to indicate by circling *how often* you felt or thought a certain way.**

Name ______________________________ Date ____________

Age ________ Gender (*Circle*): **M F** Other ______________________

0 = Never 1 = Almost Never 2 = Sometimes 3 = Fairly Often 4 = Very Often

1. In the last month, how often have you been upset because of something that happened unexpectedly?	0	1	2	3	4
2. In the last month, how often have you felt that you were unable to control the important things in your life?	0	1	2	3	4
3. In the last month, how often have you felt nervous and "stressed"?	0	1	2	3	4
4. In the last month, how often have you felt confident about your ability to handle your personal problems?	0	1	2	3	4
5. In the last month, how often have you felt that things were going your way?	0	1	2	3	4
6. In the last month, how often have you found that you could not cope with all the things that you had to do?	0	1	2	3	4
7. In the last month, how often have you been able to control irritations in your life?	0	1	2	3	4
8. In the last month, how often have you felt that you were on top of things?	0	1	2	3	4
9. In the last month, how often have you been angered because of things that were outside of your control?	0	1	2	3	4
10. In the last month, how often have you felt difficulties were piling up so high that you could not overcome them?	0	1	2	3	4

mind garden

info@mindgarden.com

www.mindgarden.com

References

The PSS Scale is reprinted with permission of the American Sociological Association, from Cohen, S., Kamarck, T., and Mermelstein, R. (1983). A global measure of perceived stress. *Journal of Health and Social Behavior, 24,* 386-396.

Cohen, S. and Williamson, G. Perceived Stress in a Probability Sample of the United States. Spacapan, S. and Oskamp, S. (Eds.) *The Social Psychology of Health.* Newbury Park, CA: Sage, 1988.

The Center for Epidemiologic Studies Depression Scale

Below is a list of the ways you might have felt or behaved. Please circle the number for each statement which best describes how often you felt or behaved this way during the past week using the following descriptions:

0. rarely or none of the time (less than 1 day)
1. some or a little of the time (1 to 2 days)
2. occasionally or a moderate amount of the time (3 to 4 days)
3. most or all of the time (5 to 7 days)

**During the past week:**	Rarely	A little	Moderate amount	Most or all the time
1. I was bothered by things that usually don't bother me.	0	1	2	3
2. I did not feel like eating; my appetite was poor.	0	1	2	3
3. I felt that I could not shake off the blues even with help from my family or friends.	0	1	2	3
4. I felt hat I was just as good as other people.	0	1	2	3
5. I had trouble keeping my mind on what I was doing.	0	1	2	3
6. I felt depressed.	0	1	2	3
7. I felt that everything I did was an effort.	0	1	2	3
8. I felt hopeful about the future.	0	1	2	3
9. I thought my life had been a failure.	0	1	2	3
10. I felt fearful.	0	1	2	3
11. My sleep was restless.	0	1	2	3
12. I was happy.	0	1	2	3
13. I talked less than usual.	0	1	2	3
14. I felt lonely.	0	1	2	3
15. People were unfriendly.	0	1	2	3
16. I enjoyed life.	0	1	2	3
17. I had crying spells.	0	1	2	3
18. I felt sad.	0	1	2	3
19. I felt that people disliked me.	0	1	2	3
20. I could not get "going."	0	1	2	3

SELF-EVALUATION QUESTIONNAIRE **STAI Form Y-1**

Please provide the following information:

Name____Date_____________S_____

Age__________________ Gender (*Circle*) **M** **F** T_____

DIRECTIONS:

A number of statements which people have used to describe themselves are given below. Read each statement and then blacken the appropriate circle to the right of the statement to indicate how you feel *right* now, that is, *at this moment*. There are no right or wrong answers. Do not spend too much time on any one statement but give the answer which seems to describe your present feelings best.

	NOT AT ALL	SOMEWHAT	MODERATELY SO	VERY MUCH SO
1. I feel calm	**1**	**2**	**3**	**4**
2. I feel secure	**1**	**2**	**3**	**4**
3. I am tense	**1**	**2**	**3**	**4**
4. I feel strained	**1**	**2**	**3**	**4**
5. I feel at ease	**1**	**2**	**3**	**4**
6. I feel upset	**1**	**2**	**3**	**4**
7. I am presently worrying over possible misfortunes	**1**	**2**	**3**	**4**
8. I feel satisfied	**1**	**2**	**3**	**4**
9. I feel frightened	**1**	**2**	**3**	**4**
10. I feel comfortable	**1**	**2**	**3**	**4**
11. I feel self-confident	**1**	**2**	**3**	**4**
12. I feel nervous	**1**	**2**	**3**	**4**
13. I am jittery	**1**	**2**	**3**	**4**
14. I feel indecisive	**1**	**2**	**3**	**4**
15. I am relaxed	**1**	**2**	**3**	**4**
16. I feel content	**1**	**2**	**3**	**4**
17. I am worried	**1**	**2**	**3**	**4**
18. I feel confused	**1**	**2**	**3**	**4**
19. I feel steady	**1**	**2**	**3**	**4**
20. I feel pleasant	**1**	**2**	**3**	**4**

© 1968, 1977 Charles D. Spielberger. All Rights Reserved.
Published by Mind Garden, Inc., www.mindgarden.com

SELF-EVALUATION QUESTIONNAIRE

STAI Form Y-2

Name____Date_________

DIRECTIONS

A number of statements which people have used to describe themselves are given below. Read each statement and then blacken in the appropriate circle to the right of the statement to indicate you *generally* feel.

	ALMOST NEVER	SOMETIMES	OFTEN	ALMOST ALWAYS
21. I feel pleasant	**1**	**2**	**3**	**4**
22. I feel nervous and restless	**1**	**2**	**3**	**4**
23. I feel satisfied with myself	**1**	**2**	**3**	**4**
24. I wish I could be as happy as others seem to be	**1**	**2**	**3**	**4**
25. I feel like a failure	**1**	**2**	**3**	**4**
26. I feel rested	**1**	**2**	**3**	**4**
27. I am "calm, cool, and collected"	**1**	**2**	**3**	**4**
28. I feel that difficulties are piling up so that I cannot overcome them	**1**	**2**	**3**	**4**
29. I worry too much over something that really doesn't matter	**1**	**2**	**3**	**4**
30. I am happy	**1**	**2**	**3**	**4**
31. I have disturbing thoughts	**1**	**2**	**3**	**4**
32. I lack self-confidence	**1**	**2**	**3**	**4**
33. I feel secure	**1**	**2**	**3**	**4**
34. I make decisions easily	**1**	**2**	**3**	**4**
35. I feel inadequate	**1**	**2**	**3**	**4**
36. I am content	**1**	**2**	**3**	**4**
37. Some unimportant thought runs through my mind and bothers me	**1**	**2**	**3**	**4**
38. I take disappointments so keenly that I can't put them out of my mind	**1**	**2**	**3**	**4**
39. I am a steady person	**1**	**2**	**3**	**4**
40. I get in a state of tension or turmoil as I think over my recent concerns and interests	**1**	**2**	**3**	**4**

© 1968, 1977 Charles D. Spielberger. All Rights Reserved.
Published by Mind Garden, Inc., www.mindgarden.com
